# Non-*fumigatus* Aspergillus Infection Associated with a Negative Aspergillus Precipitin Test in Patients with Chronic Pulmonary Aspergillosis

**DOI:** 10.1128/JCM.02018-21

**Published:** 2022-02-16

**Authors:** Keita Takeda, Junko Suzuki, Akira Watanabe, Osamu Narumoto, Masahiro Kawashima, Yuka Sasaki, Hideaki Nagai, Katsuhiko Kamei, Hirotoshi Matsui

**Affiliations:** a Center for Pulmonary Diseases, National Hospital Organization Tokyo National Hospital, Tokyo, Japan; b Division of Clinical Research, Medical Mycology Research Center, Chiba University, Chiba, Japan; University of Utah

**Keywords:** *Aspergillus fumigatus*, *Aspergillus* antibody test, sensitivity

## Abstract

Aspergillus antibody testing is key for the clinical diagnosis of chronic pulmonary aspergillosis (CPA) with high sensitivity. However, false-negative results in patients with CPA might be obtained, depending on the Aspergillus species. The aim of this study was to investigate which factors are associated with false-negative results in Aspergillus precipitin tests and whether the sensitivity of precipitin tests in CPA is influenced by Aspergillus fumigatus and non-*fumigatus* Aspergillus species. Between February 2012 and December 2020, 116 consecutive antifungal treatment-naive patients with CPA were identified and included in this retrospective chart review. Aspergillus species isolated from the respiratory tract of patients were identified by DNA sequencing. Characteristics of patients with positive and negative results for Aspergillus precipitin tests were compared. The sensitivity of the Aspergillus precipitin tests was compared between patients with A. fumigatus-associated CPA and non-*fumigatus* Aspergillus-associated CPA. A non-*fumigatus* Aspergillus species was the only factor significantly associated with negative Aspergillus precipitin test results in patients with CPA in the multivariate analysis (hazard ratio, 8.3; 95% confidence interval, 3.2 to 22.1; *P *< 0.0001). The positivity of the Aspergillus precipitin test for patients with non-*fumigatus* Aspergillus-associated CPA was lower than that for patients with A. fumigatus-associated CPA (84.8% versus 37.9%; *P *< 0.0001). These results revealed that the presence of non-*fumigatus* Aspergillus-associated CPA should be considered with a negative Aspergillus precipitin test; this finding may prevent diagnostic delay or misdiagnosis for CPA.

## INTRODUCTION

Chronic pulmonary aspergillosis (CPA) is a refractory fungal disease occurring as a complication of various pulmonary diseases (1–4); it has an estimated global prevalence of approximately 3 million (5). CPA is diagnosed based on clinical, microbiological, radiological, serological, and histopathological findings (1–3). The microbiological tests are important in the definite diagnosis of CPA (1–3). However, the culture-positive rate in CPA remains relatively low (6). When microbiological tests are negative, serological assays, particularly Aspergillus antibody testing, are key diagnostic tests with high sensitivity for CPA.

The Aspergillus precipitin test and Aspergillus-specific IgG assay are two Aspergillus antibody testing methods utilized commonly for diagnosing CPA. Among precipitin tests, the Ouchterlony double immunodiffusion technique has been performed to diagnose aspergillosis since the 1960s (7); this precipitin test is still used worldwide, although the efficacy of Aspergillus-specific IgG assays has been reported to be higher than that of precipitin tests (8, 9).

In previous studies, the sensitivity of the precipitin test has ranged from 59% to 88% in patients with CPA (8, 10). It was suggested that the false-negative results in Aspergillus antibody tests were caused by the host immune deficiency (11) and Aspergillus species (12). The causative organism associated most commonly with CPA is Aspergillus fumigatus, and the efficacy of Aspergillus antibody testing has been proven in most cases of A. fumigatus-associated CPA (8, 13). Conversely, the disease may be caused by other members of the genus Aspergillus, i.e., non-*fumigatus* Aspergillus, such as Aspergillus niger, Aspergillus tubingensis, Aspergillus welwitschiae, Aspergillus flavus, and Aspergillus terreus (14). The sensitivity of Aspergillus antibody testing for patients with non-*fumigatus* Aspergillus-associated CPA remains unknown.

The false-negative results in Aspergillus antibody tests might lead to a misdiagnosis or diagnostic delay in patients with CPA, which is a potential risk for poor prognosis due to treatment delay. Therefore, in this study, we aimed to investigate which factors are associated with false-negative results in Aspergillus precipitin tests and whether the sensitivity of precipitin tests in CPA is influenced by the species of Aspergillus, comparing between A. fumigatus and non-*fumigatus* Aspergillus species.

## MATERIALS AND METHODS

### Diagnostic criteria of CPA.

CPA was diagnosed based on chronic pulmonary symptoms lasting for a few months and thoracic imaging showing cavitation, pleural thickening, pericavitary infiltrates, a fungal ball, or parenchymal destruction or fibrosis, as specified in practice guidelines by the Infectious Diseases Society of America (2) and the joint guidelines of the European Society for Clinical Microbiology and Infectious Diseases and the European Respiratory Society (3).

### Study participants.

**FIG 1:**
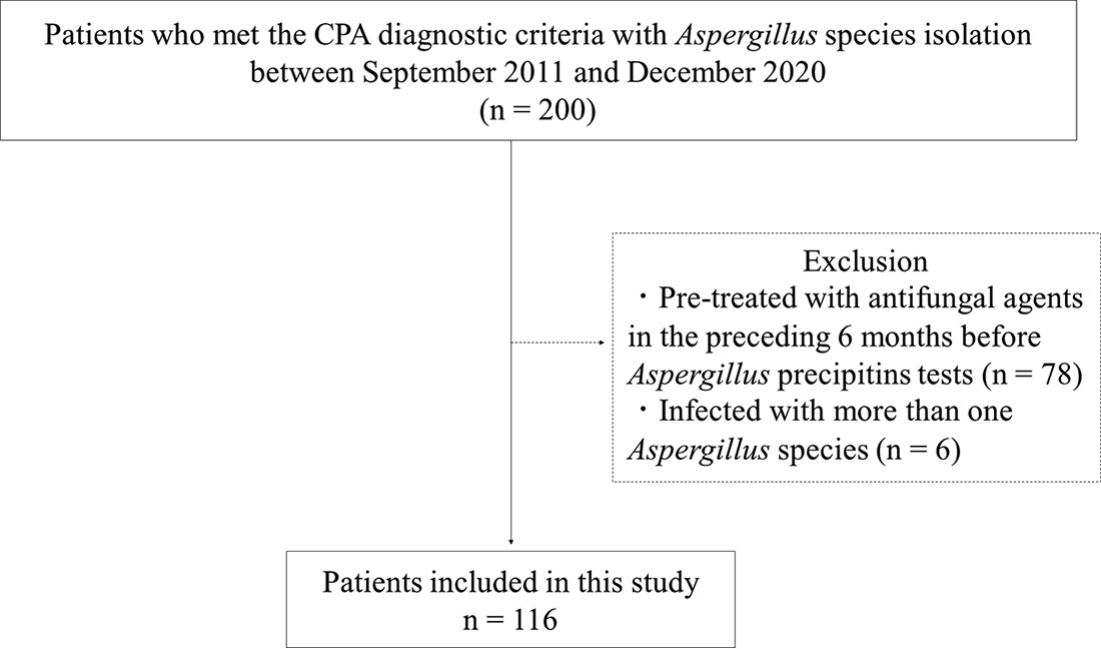
Patient selection flowchart. A total of 116 patients were included in this study after the exclusion of cases that did not fulfil the selection criteria.

This study was a retrospective chart review. The criteria for patient selection are shown in Fig. 1. Between February 2012 and December 2020, we identified 200 consecutive patients who met the CPA criteria described above, with Aspergillus species isolated from the patient respiratory tract at the NHO Tokyo National Hospital, Tokyo, Japan. A total of 116 patients with CPA were included in the study following the exclusion of (i) 78 patients who had undergone antifungal treatment in the preceding 6 months, as this treatment could possibly influence the values of Aspergillus antibodies in the precipitin test, and (ii) 6 patients who were suspected of being coinfected with more than 1 Aspergillus species within 6 months before and after CPA diagnosis to avoid confounding effects of coinfection on the antibody test.

Patient characteristics, including age; sex; underlying lung diseases; comorbidity due to diabetes mellitus, human immunodeficiency virus seropositive (HIV), malignant diseases, and atopic diseases; use of corticosteroids and/or immunosuppressants; type of CPA; overlap allergic bronchopulmonary aspergillosis; and laboratory findings were noted.

The Institutional Review Board of NHO Tokyo National Hospital (approval number 200048) approved this study and waived the requirement for written informed consent.

### Aspergillus species identification.

Lower respiratory tract samples, i.e., sputum, bronchoalveolar lavage fluid, endotracheal aspirate, and surgical samples, were cultured on Sabouraud dextrose agar (Kanto Kagaku, Tokyo, Japan) or potato dextrose agar (Kanto Kagaku), as described previously (15, 16). Aspergillus species were identified genetically using DNA sequencing of the domain 1/domain 2, internally transcribed spacer regions 1 and 2, and β-tubulin and calmodulin genes, based on previously described methods (17–19).

### Aspergillus precipitin test.

The Aspergillus precipitin test was performed within 1 month of CPA diagnosis using the FSK1 Aspergillus immunodiffusion system (Microgen, Surrey, UK), according to the manufacturer’s instructions (20). The results were recorded as “1+” to “3+” based on the precipitation arc. The results of 1+ or 2+ were recorded according to the number of precipitation arcs. The result of 3+ was recorded when the number of precipitation arcs was >2.

### Statistical analysis.

The chi-square test for frequency measurements and the *t* test for continuous variables were used to verify statistical differences in characteristics between patients with positive and negative Aspergillus precipitin test results and in the sensitivity of the Aspergillus precipitin test between patients with A. fumigatus-associated CPA and those with non-*fumigatus* Aspergillus-associated CPA. For the multivariate analysis, logistic regression analysis followed by stepwise selection (inclusion criteria, *P *< 0.25; exclusion criteria, *P *< 0.10) was used to detect factors associated with a negative result on the Aspergillus precipitin tests. A *P* value of <0.05 was considered statistically significant. Statistical analyses were performed using JMP 13.00 (SAS Institute Inc., Cary, NC, USA).

## RESULTS

### Patient characteristics.

Table 1 indicates all patient characteristics, including the comparison of patients with positive and negative Aspergillus precipitin tests. The proportion of cases caused by non-*fumigatus* Aspergillus species was significant for patients with negative Aspergillus precipitin test results (*P *< 0.0001). There were no significant differences between the two groups in terms of the other characteristics.

**TABLE 1:** Characteristics of patients with CPA and factors associated with negative Aspergillus precipitin tests^*a*^

Characteristic	Data^*b*^ for:			Univariate analysis^*c*^ *P* value	Multivariate analysis	
	All patients (*n* = 116)	CPA patients with positive Aspergillus precipitin tests (*n* = 82)	CPA patients with negative Aspergillus precipitin tests (*n* = 34)			
					Adjusted OR (95% CI)	*P* value
Age (yrs)	69.0 ± 12.0	68.7 ± 12.3	69.9 ± 11.2	0.62		
Male/female	78 (67.2)/38 (32.8)	57 (69.5)/25 (30.5)	21 (61.8)/13 (38.2)	0.52		
Aspergillus species				<0.0001		
* * Aspergillus fumigatus	87 (75.0)	71 (87.8)	16 (47.1)			
* *non-*fumigatus* Aspergillus	29 (25.0)	11 (14.1)	18 (52.9)		8.3 (3.2–22.1)	<0.0001
Underlying pulmonary diseases^*d*^				0.81		
* *Prior pulmonary tuberculosis	38 (32.8)	30 (36.6)	8 (23.5)			
* *Nontuberculous pulmonary infection	29 (25.0)	20 (24.4)	9 (26.5)			
* *Chronic obstructive pulmonary disease	24 (20.7)	15 (18.3)	9 (26.5)			
* *Interstitial lung disease	11 (9.5)	8 (9.8)	3 (8.8)			
* *Bronchiectasis	10 (8.6)	6 (7.3)	4 (11.8)			
* *History of thoracic surgery	9 (7.8)	7 (8.5)	2 (5.9)			
* *Lung cancer	6 (5.2)	5 (6.1)	1 (2.9)			
* *Others^*e*^	10 (8.6)	8 (9.8)	2 (5.9)			
Comorbidities						
* *Diabetes mellitus	11 (9.5)	8 (9.8)	3 (8.8)	>0.99		
* *HIV	0	0	0	>0.99		
* *Malignant diseases^*f*^	8 (6.9)	6 (7.3)	2 (5.9)	>0.99		
* *Atopic diseases^*g*^	11 (9.5)	9 (11.0)	2 (2.9)	0.50		
Use of corticosteroids and/or immunosuppressants	10 (8.6)	7 (8.5)	3 (8.8)	>0.99		
* *Type of CPA				0.19		
* *Simple aspergilloma	4 (3.4)	1 (1.2)	3 (8.8)			
* *Chronic cavitary pulmonary aspergillosis	83 (71.6)	59 (72.0)	24 (70.6)			
* *Chronic fibrosing pulmonary aspergillosis	18 (15.5)	13 (15.8)	5 (14.7)			
* *Subacute invasive aspergillosis	11 (9.5)	9 (11.0)	2 (5.9)			
* *Aspergillus nodule	0	0	0			
* *ABPA overlap	1 (0.9)	1 (1.2)	0	>0.99		
Laboratory findings at diagnosis						
* *White blood cell count (cells/μL)	8,400 ± 3,968	8,439 ± 3,856	8,150 ± 4,211	0.72		
* *Lymphocyte count (cells/μL)	1,281 ± 617	1,227 ± 547	1,399 ± 745	0.17		
* *Eosinophil count (cells/μL)	283 ± 479	329 ± 554	210 ± 213	0.23		
* *CRP (mg/dL)	6.3 ± 7.4	6.7 ± 7.1	4.7 ± 7.8	0.18		

a CPA, chronic pulmonary aspergillosis; HIV, human immunodeficiency virus; ABPA, allergic bronchopulmonary aspergillosis; CRP, C-reactive protein; OR, odds ratio; CI, confidence interval.

b Data are presented as mean ± SD or *n* (%).

c Comparison between CPA patients with positive and negative precipitin tests.

d Including duplicate cases.

e Including asbestosis, pneumoconiosis, bronchial asthma, and alveolar proteinosis.

f Including lung cancer and gastric cancer.

g Including bronchial asthma, atopic dermatitis, allergic rhinitis, and atopic conjunctivitis.

The presence of non-*fumigatus* Aspergillus was the only significant factor associated with negative results of the Aspergillus precipitin test in the multivariate analysis (*P *< 0.0001; hazard ratio, 8.3; 95% confidence interval, 3.2 to 22.1) (Table 1).

### Species identification.

**FIG 2:**
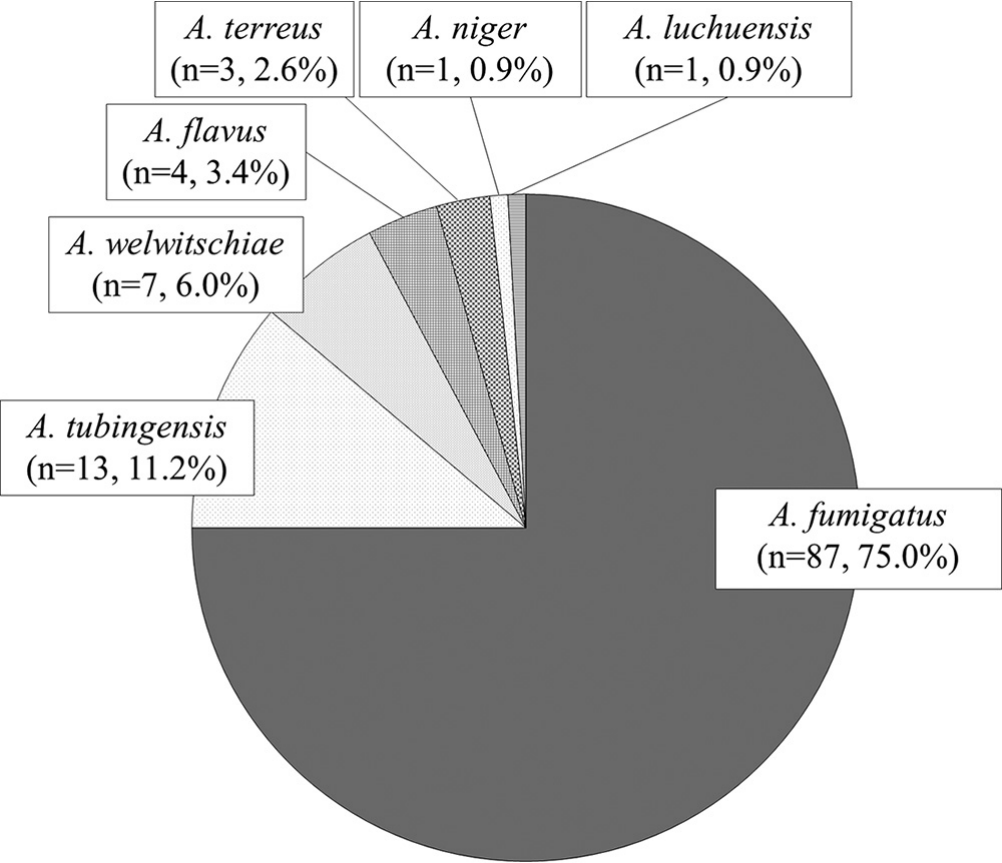
Aspergillus species isolated from the patients with chronic pulmonary aspergillosis (CPA). Aspergillus fumigatus *sensu stricto* was the most frequently isolated species, followed by A. tubingensis, *A. welwitschiae*, A. flavus, and A. terreus.

Results of Aspergillus species identification among the patients are shown in Fig. 2. Aspergillus fumigatus *sensu stricto* was the most frequently isolated species (*n* = 87, 75.0%) followed by A. tubingensis (*n* = 13, 11.2%), *A. welwitschiae* (*n* = 7, 6.0%), A. flavus (*n* = 4, 3.4%), A. terreus (*n* = 3, 2.6%), A. niger (*n* = 1, 0.9%), and Aspergillus luchuensis (*n* = 1, 0.9%).

### Results of Aspergillus precipitin tests.

Table 2 shows the results of the Aspergillus serological tests for patients with A. fumigatus-associated CPA and those with non-*fumigatus* Aspergillus-associated CPA. The positivity of the Aspergillus precipitin test for patients with non-*fumigatus* Aspergillus-associated CPA (37.9%) was lower than that for patients with A. fumigatus-associated CPA (81.6%) (*P *< 0.0001). The values of the semiquantitative analysis for the Aspergillus precipitin tests were also lower for patients with non-*fumigatus* Aspergillus-associated CPA than for patients with A. fumigatus-associated CPA (*P *< 0.0001).

**TABLE 2:** Aspergillus serological tests

Test	Data^*a*^ for:			*P* value^*b*^
	All patients (*n* = 116)	Patients with Aspergillus fumigatus-associated CPA^*c*^ (*n* = 87)	Patients with non-*fumigatus* Aspergillus*-*associated CPA (*n* = 29)	
Positive Aspergillus precipitin test	82 (70.7)	71 (81.6)	11 (37.9)	<0.0001
Semiquantitative results of Aspergillus precipitin test				<0.0001
Negative	34 (29.3)	16 (18.4)	18 (62.1)	
1+	20 (17.2)	13 (14.9)	7 (24.1)	
2+	12 (10.3)	10 (11.5)	2 (6.9)	
3+	50 (43.1)	48 (55.2)	2 (6.9)	

a Data are presented as *n* (%).

b Comparison between patients with CPA by A. fumigatus and non-*fumigatus* Aspergillus.

c CPA, chronic pulmonary aspergillosis.

### Precipitin test results based on Aspergillus species.

Table 3 shows the results of the Aspergillus precipitin test for patients according to each Aspergillus species. The positivity rates of the precipitin test for patients infected with the Aspergillus species varied from 0% to 100%.

**TABLE 3:** Aspergillus precipitin test in patients with CPA^*a*^ according to each non-*fumigatus* Aspergillus species

Test	*n* (%) by species					
	A. tubingensis (*n* = 13)	*A. welwitschiae* (*n* = 7)	A. flavus (*n* = 4)	A. terreus (*n* = 3)	A. niger (*n* = 1)	*A. luchuensis* (*n* = 1)
Positive Aspergillus precipitin test	2 (15.4)	3 (42.9)	4 (100)	2 (66.7)	0	0
Semiquantitative results						
Negative	11 (84.6)	4 (57.1)	0	0	1 (100)	1 (100)
1+	1 (7.7)	3 (42.9)	2 (50.0)	1 (33.3)	0	0
2+	0	0	1 (25.0)	1 (33.3)	0	0
3+	1 (7.7)	0	1 (25.0)	0	0	0

a CPA, chronic pulmonary aspergillosis.

## DISCUSSION

In this study, non-*fumigatus* Aspergillus infection was observed to be the cause of negative Aspergillus precipitin test results for patients with CPA. The sensitivity and semiquantitative analysis values of the Aspergillus precipitin test were significantly lower for patients infected with non-*fumigatus* Aspergillus than those for patients infected with A. fumigatus.

The Aspergillus precipitin test was positive for approximately 40% of patients with non-*fumigatus* Aspergillus-associated CPA with a low reaction level in the semiquantitative analysis. The low sensitivity of the Aspergillus precipitin test for patients with non-*fumigatus* Aspergillus-associated CPA might have occurred because the antigens detected using the test kit had been obtained from the culture of A. fumigatus (20). Nevertheless, the reactions might have occurred owing to (i) cross-reactions between A. fumigatus and other Aspergillus species (21, 22) or (ii) mixed infections with A. fumigatus (23). Although patients infected with more than one Aspergillus species were excluded from the culture study, those infected with A. fumigatus might have been included in the non-*fumigatus* Aspergillus group because of the low sensitivity of the culture tests. Aspergillus precipitin tests using antigens obtained from each Aspergillus species were not performed in this study.

In the Aspergillus antibody testing, the strength of cross-reactions between A. fumigatus and other Aspergillus species might vary among different Aspergillus species. It has been reported that cross-reactivity is higher in patients with A. flavus-associated pulmonary aspergillosis than that in patients with A. niger- and A. terreus-associated pulmonary aspergillosis (21, 22). Correspondingly, the positivity in our study was 100% in four patients with A. flavus-associated CPA. The positivity was 0 to 42.9% for Aspergillus section *Nigri*, namely, A. tubingensis, *A. welwitschia*, A. niger, and *A. luchuensis*, and 66.7% for A. terreus.

The sensitivity in patients with A. fumigatus-associated CPA was approximately 80%. In other words, approximately 20% of the tests revealed false-negative results. The reasons for the false-negative results were presumed to be as follows. (i) One reason is the sensitivity of the precipitin test, as Page et al. suggested that this sensitivity was lower than that of Aspergillus-specific IgG tests (8). The precipitin test is a subjective test dependent partly on the testers; therefore, very low reaction levels may not be detected. (ii) Another reason is early-stage infection (20), as antibodies against Aspergillus infection might develop during disease progression. Therefore, antibody tests during the early stage of CPA might give false-negative results.

Aspergillus-specific IgG assays have recently become one of the best practices for diagnosing CPA. However, there has been no study regarding the efficacy of the test in patients with non-*fumigatus* Aspergillus-associated CPA. Guo et al. suggested that Aspergillus-specific IgG might be low for patients with non-*fumigatus* Aspergillus-associated CPA (12). This low sensitivity might be because most Aspergillus-specific IgG tests use recombinant proteins produced from A. fumigatus as antigens (9). Consequently, it is necessary to verify the sensitivity of Aspergillus-specific IgG assays for patients with non-*fumigatus* Aspergillus-associated CPA by comparing them with the Aspergillus-specific IgG assays for each Aspergillus species. In addition, new diagnostic tests will be required to increase the diagnostic sensitivity for CPA patients with non-*fumigatus* Aspergillus by using a common antigen of Aspergillus species.

Disregarding Aspergillus species, it was suggested that host immunodeficiency may influence the positivity of Aspergillus antibody tests (11). In this study, to the best of our knowledge, there were no immunodeficiency factors associated with false-negative results in the Aspergillus precipitin tests. The patients were evaluated regarding diabetes mellitus, malignant diseases, and use of corticosteroids and/or immunosuppressants; in addition, white blood cell and lymphocyte counts were determined. Other immune factors, such as immunoglobulin levels, could not be evaluated in this study. Further study will be needed to assess the influence of host immunodeficiency on Aspergillus antibody tests.

Our study has the following limitations. First, the number of Aspergillus species in patients with non-*fumigatus* Aspergillus-associated CPA varied and was small. This result warrants an investigation of the sensitivity of patients with CPA to each Aspergillus species. Second, this study was a single-center analysis; a multicenter study is required to validate our findings. Third, the Aspergillus precipitin test shows subjective semiquantitative values. A quantitative analysis is required to verify the interpretation of our study.

In conclusion, the sensitivity of the Aspergillus precipitin test for patients with CPA depends on the causative Aspergillus species. To prevent diagnostic delay, the presence of non-*fumigatus* Aspergillus-associated CPA should be considered in cases of suspected CPA with a negative Aspergillus precipitin test.
